# Non-invasive evaluation of nigrostriatal neuropathology in a proteasome inhibitor rodent model of Parkinson's disease

**DOI:** 10.1186/1471-2202-11-1

**Published:** 2010-01-05

**Authors:** Anthony C Vernon, Saga M Johansson, Michel M Modo

**Affiliations:** 1 Department of Neuroscience, Kings College London, Centre for the Cellular Basis of Behaviour, The James Black Centre, 125 Coldharbour Lane, London SE5 9NU, UK

## Abstract

**Background:**

Predominantly, magnetic resonance imaging (MRI) studies in animal models of Parkinson's disease (PD) have focused on alterations in T_2 _water ^1^H relaxation or ^1^H MR spectroscopy (MRS), whilst potential morphological changes and their relationship to histological or behavioural outcomes have not been appropriately addressed. Therefore, in this study we have utilised MRI to scan *in vivo *brains from rodents bearing a nigrostriatal lesion induced by intranigral injection of the proteasome inhibitor lactacystin.

**Results:**

Lactacystin induced parkinsonian-like behaviour, characterised by impaired contralateral forelimb grip strength and increased contralateral circling in response to apomorphine. T_2_-weighted MRI, 3-weeks post-lesion, revealed significant morphological changes in PD-relevant brain areas, including the striatum and ventral midbrain in addition to a decrease in T_2 _water ^1^H relaxation in the substantia nigra (SN), but not the striatum. Post-mortem histological analyses revealed extensive dopaminergic neuronal degeneration and α-synuclein aggregation in the SN. However, extensive neuronal loss could also be observed in extra-nigral areas, suggesting non-specific toxicity of lactacystin. Iron accumulation could also be observed throughout the midbrain reflecting changes in T_2_. Importantly, morphological, but not T_2 _relaxivity changes, were significantly associated with both behavioural and histological outcomes in this model.

**Conclusions:**

A pattern of morphological changes in lactacystin-lesioned animals has been identified, as well as alterations in nigral T_2 _relaxivity. The significant relationship of morphological changes with behavioural and histological outcomes in this model raises the possibility that these may be useful non-invasive surrogate markers of nigrostriatal degeneration *in vivo*.

## Background

Parkinson's disease (PD) is a progressive neurodegenerative movement disorder characterised by a selective vulnerability and degeneration of dopaminergic (DA) neurons in the substantia nigra pars compacta (SNc) [1]. This is accompanied by formation of eosinophilic cytoplasmic inclusions in remaining neurons, termed Lewy bodies (LB), composed primarily of fibrillar aggregates of α-synuclein [2].

Degeneration of DA neurons in the SNc results in significant depletion of striatal dopamine levels, which can be readily visualised in PD patients using Positron Emission Tomography (PET) in combination with specific radiotracers, such as 18-fluorodopa (^18^F-DOPA) [3]. However, whilst there is abundant PET data for PD, the results of magnetic resonance imaging (MRI)-morphometric studies regarding the basal ganglia (BG) in PD patients are still relatively scarce and inconsistent [4]. Indeed, brain atrophy is not routinely ascribed to human idiopathic PD (iPD), particularly in cognitively intact patients [5]. In early-stage iPD, both normal [6,7] and partial reductions [8-10] in grey matter volume (GMV) of the BG have been reported [4]. Similarly, voxel-based morphometry (VBM) studies have reported significant morphological changes, including atrophy of the head of the left caudate nucleus and cortical changes in both early and advanced iPD, which correlated robustly with clinical symptoms [4,11]. Furthermore, significant asymmetrical hypertrophy of the lateral ventricles (LV) has been reported, which also correlated robustly with patient disability scores on the Unified Parkinson's Disease Rating Scale (UPDRS) [12]. Importantly, patterns of morphometric change which can be readily detected by MRI are already utilised clinically to aid differential diagnosis of iPD from other forms of parkinsonism, including the α-synucleinopathy multiple system atrophy (MSA) and the tauopathy progressive supranuclear palsy (PSP) [13,14].

In addition, it has been widely reported that T_2 _water proton relaxivity rates are decreased in pathologically relevant areas in iPD patients, including the SNc and putamen, which has been ascribed to regional iron accumulation [15-18]. Interestingly, a significant relationship between T_2 _relaxivity, iron accumulation and clinical symptoms has been described. Alterations in T_2 _relaxivity may therefore represent a surrogate marker for PD progression, although only cross-sectional studies have been performed to date [19,20]. By contrast, the possibility that morphometric changes, or a combination of this with alterations in T_2 _relaxation time may be used to non-invasively monitor disease progression in PD remains unclear [21].

Crucial to a more mechanistic understanding of pathology and the evaluation of novel treatments for PD are animal models that reflect important aspects of the clinical manifestation of the disease. Foremost of all, rodent toxin-based models of PD, although subject to limitations, have provided useful insights into the pathophysiology of iPD [22]. Combining such animal models with non-invasive imaging, such as MRI, offers a powerful tool with which to investigate potential dynamic morphological changes due to degeneration of the nigrostriatal system, which may lead to identification of surrogate markers of disease progression [23,24]. Importantly, detailed anatomical or relaxivity information can be correlated directly with behavioural phenotypes, raising the possibility that MRI could provide non-invasive surrogate markers predictive of the degree of functional impairment in individual animals [25].

Previous studies have elegantly demonstrated the application of MRI to animal models of PD [26-31]. Primarily however, these studies have focused on alterations in T_2 _relaxation or ^1^H MR spectroscopy (MRS) and more recently diffusion tensor imaging (DTI) [32] and manganese enhanced MRI (MEMRI) [33]. In contrast, potential morphological changes and their relationship to histological or behavioural outcomes have not been appropriately addressed. Therefore, in this study we have utilised MRI to scan *in vivo *brains from rodents bearing a nigrostriatal lesion induced by an intranigral injection of the proteasome inhibitor lactacystin. This relatively recently developed model has been suggested to be a novel, pathologically relevant model of PD [34-36], which has already been used with some success in neuroprotection studies [35-38]. Our primary aim in this study was to investigate whether morphological and T_2 _relaxation times are altered in PD-relevant brain areas in this new experimental model of PD and to determine how these are related to behavioural and histological outcomes.

## Results

### Behavioural testing

**Figure 1:**
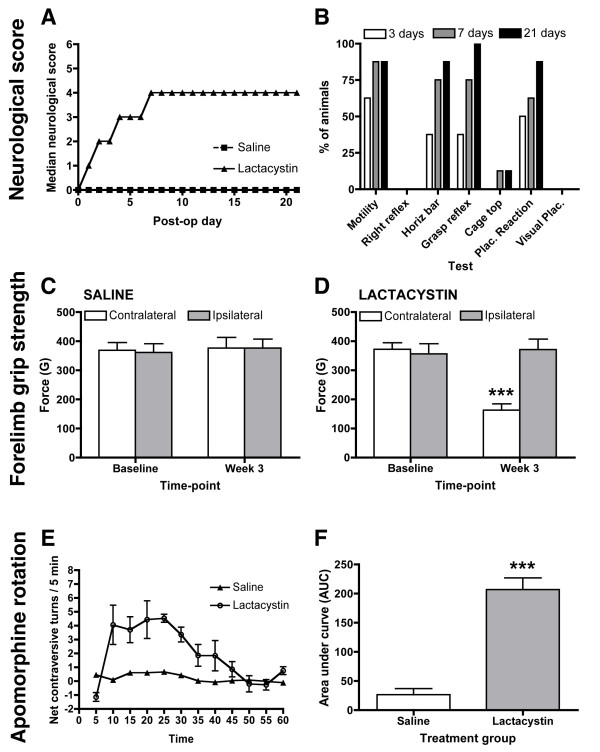
**Assessment of neurological and behavioural deficits**. (A) Neurological scoring (B) Percentage of lactacystin-lesioned animals that are impaired in each of the neurological score tests at days 3, 7 and 21. (C) No significant reduction in forelimb grip strength was observed in saline-injected controls. By contrast in lactacystin-lesioned animals (D) forelimb grip strength was significantly reduced in the contralateral forelimb at 1 and 3 weeks post-lesion. Data shown are mean forelimb grip force (g) ± SEM *p < 0.01 saline vs. lactacystin. (C) Lactacystin-lesioned animals displayed significantly increased rotational asymmetry in response to apomorphine challenge (0.1 mg/kg s.c.), Data shown are mean area under curve (AUC) ± SEM ***p < 0.001 saline vs. lactacystin.

Lactacystin-lesioned animals, but not controls, initially developed gross neurological deficits progressively, as shown by a gradual worsening in neurological score (Figure 1A). Lactacystin-lesioned animals consistently showed deficits in spontaneous motility, which may be a reflection of developing bradykinesia in these animals. Lesioned animals also demonstrated clear deficits in the horizontal bar, grasping reflex and placing reaction tests, suggesting defects in skilled forelimb use. Lesioned animals showed only moderate deficits in the cage top test and did not show any deficits in righting reflex or visual placement test (Figure 1B). Consistent with the initial progressive increase in global neurological score between 3 and 7 days, the percentage of lactacystin-lesioned animals that displayed deficits in these tests increased in this period (Figure 1B). Modest further increases in the percentage of animals displaying these deficits were observed between days 7 to 21 post-lesion (Figure 1B). However, this likely reflects inter-animal variation in lesion size and not progressive lesion development, as global neurological scores remain static (Figure 1A,B). Qualitatively, lactacystin-lesioned animals, but not controls, also developed ipsiversive postural deficits and demonstrated spontaneous circling behaviour ipsilateral to the lesioned hemisphere. Importantly, neurological scores became static by 7 days post-lesion, and did not increase further until sacrifice 3 weeks post-lesion (Figure 1A).

To determine if lactacystin-lesioning affects forelimb use, performance was assessed in the grip strength measure (GSM) test. Baseline measures of grip strength were established prior to surgery with no significant difference in average grip force observed for either forelimb in either group (Figure 1C,D). No significant deficits in grip strength between the contralateral and ipsilateral forelimbs were observed in saline-injected controls (Figure 1C). By contrast, 3-weeks post-surgery, lactacystin-lesioned animals displayed a significant decrease in the grip strength of the contralateral compared to the ipsilateral forelimb (*p *< 0.001; Figure 1D). No significant differences in the grip strength of the ipsilateral limb were observed between lactacystin-lesioned animals and controls at any time-point (Figure 1C,D). Furthermore, three-weeks post-surgery, lactacystin-lesioned animals, but not saline controls, displayed significantly increased net contraversive rotations following an apomorphine challenge (*p *< 0.001, Figure 1E, F). Lactacystin-lesioned animals displayed an average net contraversive turns of >5 turns/min. This activity increased steadily after 5 min and was maintained for approximately 35 minutes, at which point locomotor activity decreased back to baseline (Figure 1E). No spontaneous recovery of either neurological score or behaviour was observed during the 3 weeks of observation in lactacystin-lesioned animals.

### Brain regional volumes

**Figure 2:**
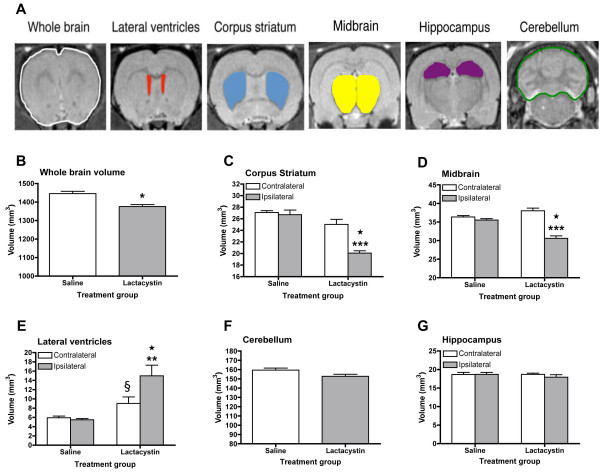
**(A) Representative T2W MR images from saline-injected control subject with sample ROIs utilised for quantitative volumetric analysis of individual brain regions**. (B-G) Bar graphs of regional brain volumetric data measured on *in vivo *T2W anatomical MRI scans acquired from saline and lactacystin-injected animals 3 weeks post-lesion. (B) Whole brain volume, (C) corpus striatum, (D) ventral midbrain, (E) lateral ventricles, (F) cerebellum and (G) hippocampal formation. Data are expressed as mean volume mm^3 ^± s.e.m. *p < 0.05, **p < 0.01; ipsilateral vs. contralateral hemisphere; *p < 0.01; ipsilateral hemisphere of lactacystin vs. saline-injected; §*p *< 0.05 contralateral hemisphere of saline vs. lactacystin-injected.

Sample ROI used to obtain volumetric data are shown in Figure 2A and the anatomical criteria used to define individual brain structures are shown in Table 1. There was a significant reduction in whole brain volume (WBV) when comparing lactacystin-lesioned animals and saline controls (1436 ± 18 vs. 1375 ± 11 mm^3^; *p *< 0.05; Figure 2B). Measurements of individual brain structures revealed numerous regional volume changes in lactacystin-lesioned animals, but not saline controls. Indeed, the ipsilateral striatum (STR) was found to be significantly smaller relative to the non-injected contralateral hemisphere in lactacystin-lesioned animals (20.04 ± 0.93 vs. 24.93 ± 0.40 mm^3^; *p *< 0.001; Figure 2C) and the ipsilateral hemisphere of saline controls (20.04 ± 0.40 vs. 26.70 ± 0.79 mm^3^; *p *< 0.001; Figure 2C). In addition, the ipsilateral ventral midbrain (VM) was strikingly deformed with a concomitant increase in the surrounding cerebrospinal fluid (CSF) space. Quantitative measurement revealed the ipsilateral VM to be significantly smaller in lesioned animals compared to both the non-injected contralateral hemisphere (38.03 ± 0.69 vs. 30.61 ± 0.66 mm^3^; *p *< 0.001; Figure 2D) and the ipsilateral hemisphere of saline control animals (35.51 ± 0.39 vs. 30.61 ± 0.66 mm^3^; *p *< 0.001; Figure 2D).

**Table 1:** Anatomical criteria for volume measurements.

Brain region	Anatomical criteria for measurement
Whole Brain	Base of the olfactory bulb to last slice containing cortex
Lateral ventricles	Defined from brain tissue by intense contrast of CSF
Corpus striatum	Defined with reference to corpus callosum, external capsule, anterior comissure and lateral ventricles
Midbrain	Defined with reference to dorsal hippocampal formation
Hippocampus	Defined with reference to corpus callosum and external capsule
Cerebellum	Defined with reference to 4^th ^ventricle and brainstem as a guide

Abbreviations: AC = anterior commissure, CC = corpus callosum, EC = external capsule

Notably, the ipsilateral lateral ventricles (LV) were significantly enlarged in lactacystin-lesioned animals compared to both the non-injected contralateral hemisphere (14.80 ± 2.30 vs. 9.02 ± 1.38 mm^3^; *p *< 0.01; Figure 2E) and the ipsilateral hemisphere of control animals (14.98 ± 2.30 vs. 5.46 ± 0.29 mm^3^; *p *< 0.01; Figure 2E). The contralateral LV in lactacystin lesioned animals was also significantly enlarged compared to saline controls (9.02 ± 1.38 vs. 5.92 ± 0.36 mm^3^; *p *< 0.05; Figure 2E).

**Figure 3:**
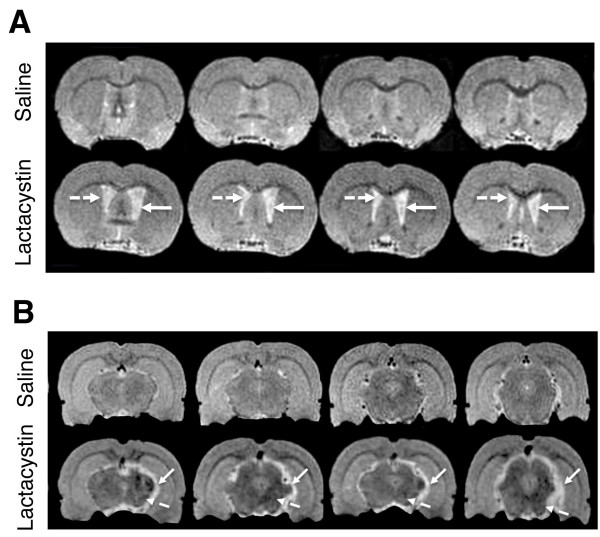
**Representative T2W MR images through the striatum (panel A) and midbrain containing the substantia nigra (SN; panel B) to illustrate regional brain atrophy in saline and lactacystin-injected animals**. Clear hypertrophy of both the ipsilateral (solid arrows, A) and contralateral (dashed arrows, A) LV can be observed in lactacystin-lesioned animals, compared to saline controls. In the midbrain (panel B) the sub-cortical area containing the SN in the ipsilateral hemisphere appears clearly deformed compared to the intact contralateral hemisphere (solid arrows). Note also the increased CSF signal, suggestive of a volume change in this region. In addition, areas of T_2 _hypointensity are observed in the SN, but also in extra-nigral nuclei (dashed arrows).

No significant volume changes were observed in either the absolute volume of the cerebellum (CB) (Figure 2F) or for the hippocampal formation (HF) in either hemisphere, when comparing saline and lactacystin-injected animals (Figure 2F). These data are illustrated in representative MR images in Figure 3A, where clear hypertrophy of the LV can be observed (Figure 3A) as well as the striking deformation of the ipsilateral VM and corresponding increase in CSF space (Figure 3B). With the exception of the contralateral LV, no significant differences were observed for any brain region when comparing the contralateral hemisphere of saline and lactacystin-injected animals.

### T_2 _signal intensity measurements

**Figure 4:**
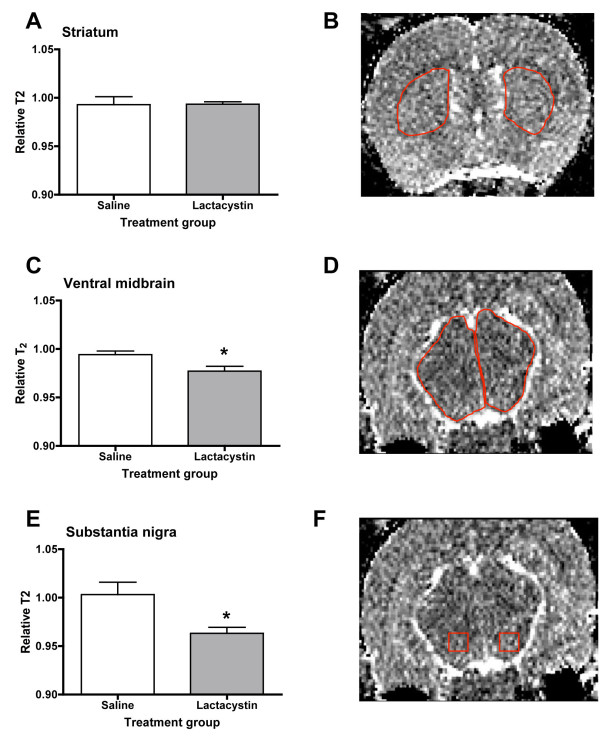
**Bar graphs of T_2 _relaxivity measured from parametric T_2 _maps generated from T2W MRI scans acquired 3 weeks post-lesion from saline and lactacystin-injected animals**. (A) No significant difference in T_2 _was observed in the striatum between saline and lactacystin-injected animals. By contrast, moderate, but significant reductions in T_2 _were observed in the ventral midbrain (C) and substantia nigra (E) of lactacystin-injected animals compared to saline controls. Example ROIs used to generate quantitative T_2 _data are shown for striatum in (B), ventral midbrain (D) and substantia nigra (F), respectively. Data shown are the ratio of T_2 _between the contralateral and ipsilateral hemispheres ± SEM for saline and lactacystin-injected animals, respectively. *p < 0.05; saline vs. lactacystin.

In lactacystin-lesioned animals, but not saline controls, visual inspection of T2W images revealed no visible areas of T_2 _signal change in the striatum in either hemisphere between saline and lactacystin-lesioned animals (Figure 3A). In contrast, areas of T_2 _hypointensity could be clearly observed in the VM, particularly in an area approximate to the location of the SN (Figure 3B). Notably, however, areas of T_2 _hypointensity were also observed in extra nigral regions throughout the ipsilateral VM (Figure 3B). These data are supported by quantitative ROI-based T_2 _measurements, which revealed no significant alterations in striatal T_2 _between hemispheres in either lactacystin-lesioned animals or saline controls (Figure 4A, B). By contrast, a moderate, but significant, decrease in T_2 _relaxivity was observed in the ipsilateral ventral midbrain and SN of lactacystin-lesioned animals, relative to the non-injected contralateral hemisphere and the ipsilateral SN of saline-injected controls (Figure 4C-F).

### Post-mortem histology

**Figure 5:**
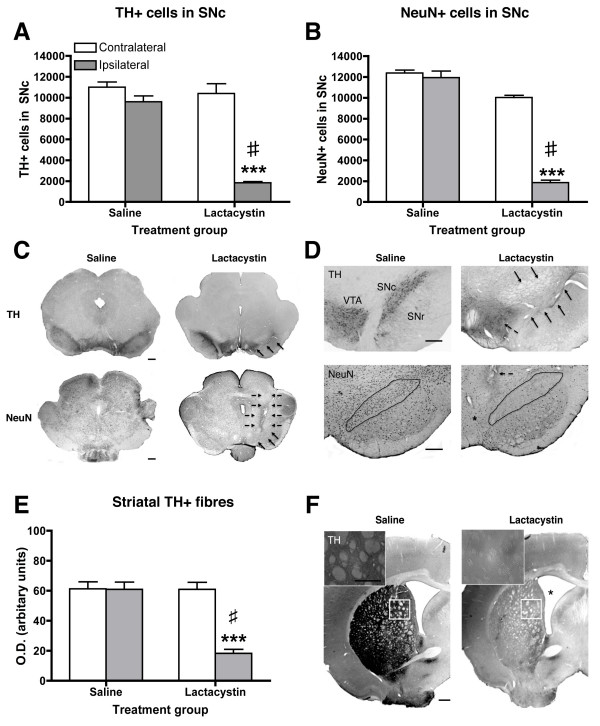
**Histological assessment of nigrostriatal integrity 3 weeks post-lesion**. Quantification by unbiased stereology revealed intranigral injection of lactacystin resulted in a marked decrease in (A) the number of TH+ cell bodies in the ipsilateral SNc, mirrored by a clear reduction in (B) NeuN+ cells. ***p < 0.001; contralateral vs. ipsilateral hemisphere, #*p *< 0.01; ipsilateral hemisphere of lactacystin vs. saline controls. (C, D) Representative photomicrographs of TH+ staining in saline and lactacystin-lesioned animals. Note the extensive loss of TH+ and NeuN+ cells in the ipsilateral SNc, but also in the VTA, SNr and extranigral nuclei in proximity to the injection tract of lactacystin-injected animals (arrows) (Magnification (C) × 1.5, scale bars 500 μm; (D) ×4, scale bars 200 μm). Concomitant with nigral cell body degeneration, a substantial dennervation of TH+ fibres was observed in the ipsilateral striatum of lactacystin-lesioned animals quantified by optical densitometry (E) and illustrated by a representative photomicrographs in (F) (magnification ×1.5, scale bar 500 μm; insets ×40, scale bar 20 μm). ***p < 0.001; contralateral vs. ipsilateral hemisphere, #*p *< 0.01; ipsilateral hemisphere of lactacystin vs. saline controls.

To validate the origin of MRI signal changes and to examine how these are associated with nigrostriatal pathology, post-mortem immunohistological analyses were performed on the nigrostriatal system. Unbiased stereology cell counts revealed that intranigral injection of lactacystin resulted in an 81.4 ± 1.1% reduction of TH-positive (+) cells in the ipsilateral substantia nigra pars compacta (SNc) compared to the intact contralateral hemisphere (10,030 ± 214 vs. 1861 ± 219; *p *< 0.001; Figure 5A). This was mirrored by an 82.2 ± 2.0% reduction in NeuN-positive (+) cells (10,380 ± 934 vs. 1825 ± 236; *p *< 0.001; Figure 5B), strongly suggesting cell death of A9 DA neurons rather than down-regulation of TH expression in atrophic neurons. No significant differences were observed in either TH+ or NeuN+ cell counts between the contralateral and ipsilateral hemispheres in saline-injected controls or between the contralateral hemispheres in lactacystin-lesioned animals and saline controls (Figure 5A, B). Notably however, the pars reticulata of the SN (SNr) and the VTA were not free from the direct toxicity of lactacystin. Indeed, qualitatively, extensive loss of both TH+ and NeuN+ cells was observed in these regions when compared to saline controls (Figure 5C, D). Microscopic analysis of extra-nigral nuclei in the ipsilateral VM revealed additional areas of neuronal loss due to the direct toxicity of lactacystin, including the red nucleus, immediately supranigral to the SNc and in numerous nuclei in the VM adjacent to the needle injection tract. These data are illustrated by representative photomicrographs in Figure 5C and 5D.

**Figure 6:**
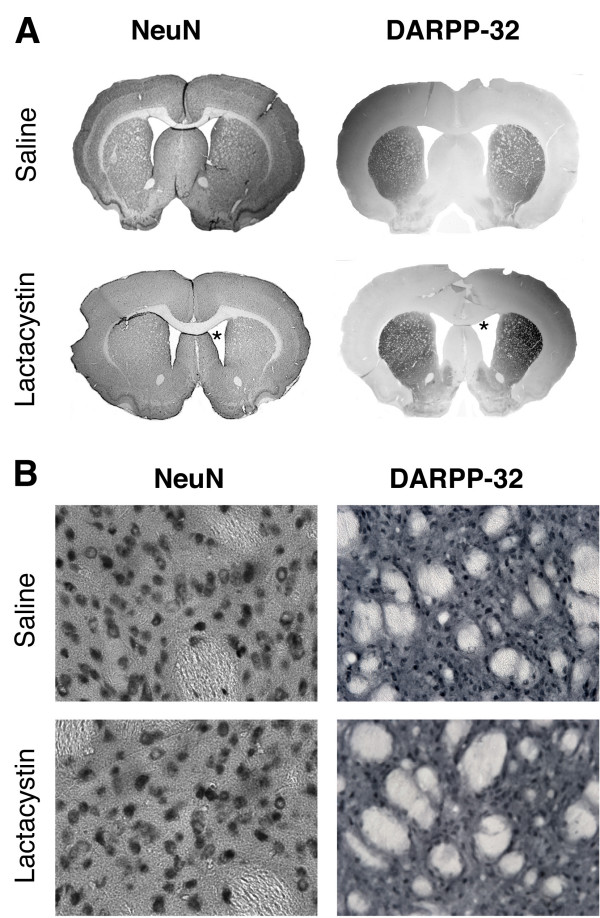
**Lactacystin lesioning does not result in loss of striatal medium spiny neurons**. (A) Whole brain photomicrogaphs of NeuN and DARPP-32+ cells in the striatum of saline and lactacystin lesioned animals (×1.5 magnification, scale bar = 500 μm). (B) Higher magnification of NeuN+ and DARPP-32+ cells in the striatum of saline and lactacystin injected animals, respectively. (×40 magnification, scale bar = 20 μm). Note the enlarged ventricles in lactacystin-injected animals (asterisks in A).

Consistent with loss of nigral TH/NeuN+ cell bodies, densitometry analysis revealed a 69.6 ± 2.5% reduction of TH+ fibre density in the ipsilateral striatum, relative to the non-injected contralateral striatum of lactacystin-lesioned animals (61.29 ± 1.99 vs. 18.61 ± 1.54; *p *< 0.001; Figure 5E, F) confirming significant loss of DA axon terminals. No significant differences were observed in TH+ fibre optical density in the contralateral striatum when comparing lactacystin-lesioned animals and saline controls, or between the contralateral and ipsilateral striatum in saline-injected animals (Figure 5E, F). Qualitatively, however, there was no apparent loss of either NeuN+ or DARPP-32+ cells in the ipsilateral striatum of lactacystin-lesioned animals, suggesting γ-amino-butyric-acid (GABA-ergic) medium spiny neurons in the STR may be preserved (Figure 6A,B).

**Figure 7:**
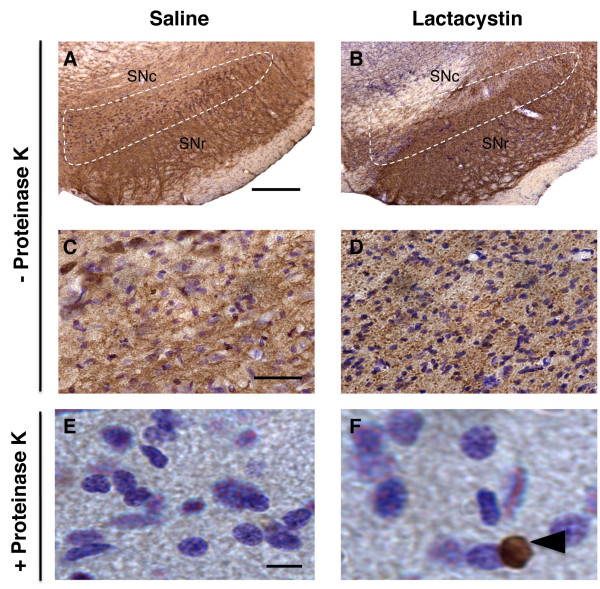
**(A-D) α-synuclein immunohistochemistry reveals the presence of soluble aggregates of α-synuclein in the substantia nigra of lactacystin-lesioned animals compared to saline controls (panels A, B: ×4 magnification, scale bars = 200 μm; panels C, D: ×40 magnification, scale bars = 20 μm)**. Note the diffuse pattern of staining in control animals compared to the punctate labelling observed in lactacystin-lesioned animals, indicative of aggregation. (E-F) Pre-treatment with proteinase K (10 μg) reveals the presence of insoluble perinuclear α-synuclein aggregates in the SNc of lactacystin-injected animals, but not saline controls (×60 magnification, scale bars = 10 μm).

In the SNc of lactacystin-lesioned animals, aggregation of α-synuclein was observed, compared to the diffuse staining in the SNc of control animals (Figure 7A-D). Pre-treatment with proteinase K allowed selective visualisation of insoluble α-synuclein deposits and abolished immunostaining for endogenous soluble α-synuclein. This revealed focal cytoplasmic proteinase-K resistant α-synuclein immunoreactive inclusions in a small proportion of cells in the SNc of lactacystin-lesioned animals, which were not present in saline controls (Figure 7E, F).

**Figure 8:**
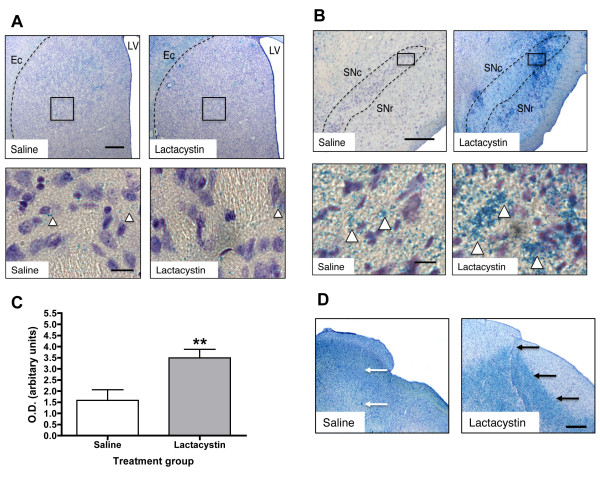
**(A) Prussian blue and cresyl violet histology reveals no apparent iron accumulation in the striatum of both saline and lactacystin-lesioned animals (arrowheads in A) (×2.5 magnification, scale bars = 300 μm; insets × 40 magnification, scale bars = 20 μm)**. (B) By contrast, extensive iron deposits are observed in the SNc of lactacystin lesioned animals but not saline controls (arrowheads in B); (×2.5 magnification, scale bars = 300 μm; insets ×40 magnification, scale bars = 20 μm). Note that iron deposition is not confined to the SN but is also present in extra-nigral regions (C) Densitometry measurements confirmed the increased in iron deposition in the SN of lactacystin-lesioned animals compared to saline controls. Data shown are mean optical density values (arbitrary units) ± SEM. **p < 0.01; saline vs. lactacystin. (D) Iron deposits were also observed in proximity to the needle-tract of lactacystin-lesioned animals, but not saline controls (arrows in D) (×2.5 magnification, scale bar = 300 μm). Interestingly, iron accumulation appears to overlap with areas of neuronal loss in the midbrain of lactacystin-lesioned animals as shown in Figure 5D.

Prussian blue (PB) histology revealed minimal iron deposits in the striatum of both lactacystin and saline-injected animals (Figure 8A). By contrast, PB histology revealed clear and extensive iron accumulation in the ipsilateral SNc of lactacystin-lesioned animals compared to saline controls (Figure 8B). In support of this, densitometry analysis exposed a significant increase in iron accumulation in the ipsilateral SNc of lactacystin lesioned animals compared to saline controls (Figure 8C). Iron deposits were, however, also observed in the SNr and in extra-nigral nuclei in the ventral midbrain, near to the injection tract, as well as in the contralateral hemisphere of lesioned animals compared to saline controls. (Figure 8B, D).

### Relationships between *in vivo *MRI, behaviour and post-mortem histology

To determine whether *in vivo *MRI measurements are linked to behavioural and histological outcomes in this model, we correlated MRI- measurements with behavioural impairments and histological measures of nigrostriatal damage in this model (Tables 2 and 3, respectively). Significant negative correlations were found between apomorphine rotation and WBV, ipsilateral STR and VM volumes (Table 2), whilst LV hypertrophy correlated positively with apomorphine rotations. By contrast, only ipsilateral STR volume was significantly associated with decreased contralateral forelimb grip strength. No significant relationships were observed between either cerebellar volume or hippocampal volume and any behavioural measure. In addition, significant associations were found between the number of TH+ and NeuN+ cells in the ipsilateral SNc with changes in WBV, ipsilateral VM and STR volume. Interestingly, a significant relationship was found between TH+ fibre density and ipsilateral STR volume. In addition, these histological measures all correlated robustly with LV hypertrophy. There was no significant link between any histological measure and HF or CB volume.

**Table 2:** Two-tailed Pearson correlations for all subjects between specific brain region volumes derived from MRI measurements, behavioural deficits and histological measurements.

Regional MRI volume	Apomorphine rotations	Forelimb grip strength	TH+ cells	NeuN+ cells	TH+ fibres
WBV	-.767**	.420	.832*	.718*	. 821**
VM	-.955**	.599	.980**	.868**	.861**
STR	-.746**	.666*	.798*	.868**	.715**
LV	.811**	-.467	-.775*	-.730*	-.713*
HPC	.489	-.211	-.496	-.312	-.398
CB	.025	-.339	.081	-.083	.034

*p < 0.05, **p < 0.01.

**Table 3:** Two-tailed Pearson correlations for all subjects between MRI volume measurements of individual brain regions and whole brain volume. *p < 0.05, **p < 0.01.

T_2 _signal intensity	Apomorphine rotations	Forelimb grip strength	TH+ cells	NeuN+ cells	TH+ fibres
SN	-.158	.405	.467	.159	.452
STR	-.044	-.076	.327	-.341	-.240

No significant associations could be found between ipsilateral SN T_2 _values any behavioural measure at 3 weeks post-lesion (Table 3). In addition, no significant associations could be found between SN T_2 _values and the number of surviving TH+ and NeuN+ cells in the SN, or the density of TH+ fibres in the striatum. However, a significant relationship could be observed between the degree of iron accumulation and T_2 _values in the SN (*r *= -.793; *p *< 0.01). No significant relationships between striatal T_2 _values and behavioural or histological outcomes were observed. For completeness, *ex vivo *post-mortem histological measures were also correlated with behavioural outcomes in this model. As expected, there were strong correlations with the number of TH+ cells in the ipsilateral SNc and both apomorphine rotations (*r *= -.947; *p *< 0.01) and forelimb grip strength (*r *= .732; *p *< 0.05). The number of NeuN+ cells in the ipsilateral SNc also strongly correlated with both apomorphine rotations (*r *= -.882; *p *< 0.01) and forelimb grip strength (*r *= .829; *p *< 0.05). Similarly, highly significant relationships were observed between TH+ cell number and NeuN+ cell number in the ipsilateral SNc (*r *= .932; *p *< 0.01) and TH+ fibre density in the striatum (*r *= .943; *p *< 0.01).

## Discussion

Using *in vivo *T2-weighted (T2W) MRI, we have been able to detect for the first time a pattern of morphological changes in the brains of rodents bearing a unilateral lesion of the nigrostriatal system induced by intranigral injection of a proteasome inhibitior. We observed a moderate, but significant global reduction in brain volume, accompanied by a striking deformation of the ipsilateral VM, shrinkage of the ipsilateral striatum and hypertrophy of the LV. Although the presence of BG atrophy in PD patients is a matter of controversy [6,7] these data correspond robustly with recent published data from clinical neuroimaging studies in iPD patients. Indeed, morphological changes, including decreased striatal volume and LV hypertrophy have been reported [4,10-12]. Nevertheless, midbrain atrophy is commonly detected in MRI from patients with atypical PD, such as MSA or PSP [13,14]. No changes were observed in either cerebellar or hippocampal volume. We further observed a slight, but significant decrease in T_2 _relaxivity in the SN, consistent with previous reports of this phenomenon in iPD patients [15-18]. However, we could not find any change in T_2 _in the STR in this model. In addition to these MRI signal changes, lactacystin-lesioned animals displayed robust parkinsonian-like behavioural defects and classical nigrostriatal pathology, including α-synuclein aggregation in the SN, consistent with previous reports describing the development of this rodent pre-clinical model [34-36].

### Relationship between morphological changes detected by MRI with behavioural and histological outcomes

Examination of the relationship between MRI signal changes and behavioural outcomes revealed that volumetric changes in the ipsilateral VM, striatum and LV were significantly correlated to apomorphine rotation, whilst only ipsilateral striatal volume was significantly associated with impaired grip strength in this model. These data suggest volumetric MRI measurements of these areas could be predictive of the degree of some, but not all, functional impairments in this model, consistent with human studies [4,12]. These preliminary data, albeit in a small number of subjects, raise the possibility that these volumetric *in vivo *MRI measures may be useful surrogate markers of disease progression in this model. In addition, previous studies have demonstrated that lactacystin-lesioned animals also show significantly impaired performance in the accelerating rotarod test and overall locomotor activity [36]. Thus it may be interesting in future studies to additionally examine the relationship between MRI volumetric changes and performance in these motor tasks in lactacystin-lesioned animals, which may provide further evidence for MRI volumetric changes in this model as surrogate markers of nigrostriatal dysfunction. Importantly, however, repetition of this analysis in a larger group of subjects and comparison to established pre-clinical models of PD (6-OHDA/MPTP) are required to fully establish the reliability and specificity of these surrogate markers.

In addition, post-mortem validation of MRI signal changes is crucial before these can be accepted as surrogate markers of disease progression. An important corollary to this is that lactacystin can induce both selective damage to DA neurons or more widespread cell loss. Indeed, *in vitro*, lactacystin has been reported to induce a significant loss of only DA neurons [39] or both DA and GABAergic neurons [40,41]. In contrast, *in vivo*, intrastriatal injection of lactacystin (10 μg) induced a general neuronal toxicity in the striatum [42]. Importantly, in the current study, volume decreases were significantly correlated with the number of surviving TH+ and NeuN+ cell numbers in the ipsilateral SN, but also the density of TH+ fibres in the ipsilateral striatum. However, lactacystin not only induced neuronal loss in the pars compacta and pars reticulata of the SN, but also in the VTA, a nucleus that is relatively preserved in human iPD [1]. Thus, whilst the morphological MRI changes observed in the current study are certainly linked to a specific loss of A9 DA neurons, at least in part, we cannot exclude the possibility that these are also due to an additional loss of other vulnerable neuronal populations.

Additionally, focal areas of neuronal loss were observed throughout the ipsilateral VM along the needle injection tract, consistent with previous reports [42]. This phenomenon most likely explains the extensive deformation of the ipsilateral VM observed on the MRI scans. Thus, although volumetric MRI measures were strongly associated with both behavioural and *ex vivo *histological measures, their use as surrogate markers of nigrostriatal damage might be limited, since volumetric changes in these regions do not discriminate between damage to the nigrostriatal or other neuronal populations in the midbrain in this model.

Although TH+ fibre density was dramatically reduced in the ipsilateral striatum of lactacystin-lesioned animals, qualitatively, no gross loss of either NeuN+ or DARPP-32+ cells was apparent. Thus, striatal medium spiny neurons (MSN) appear to be preserved in this model. Interestingly, TH+ fibre loss and dopamine depletion are reported to be associated with synaptic remodelling and dendritic changes in striatal MSN in 6-OHDA lesioned rodents [43,44]. These data are consistent with reports of decreased MSN dendrite spine number and length in the striatum of post-mortem PD brains [45]. Conceivably therefore, decreased striatal volume could be attributed to TH+ fibre loss and subsequent dendritic remodelling. It is tempting to speculate therefore that striatal volumetric change may be a conserved marker of nigrostriatal degeneration across pre-clinical models of PD and perhaps human iPD, which importantly, correlates with behavioural and clinical outcomes [4]. However, investigation of volumetric MR changes in established PD models that induce selective nigrostriatal DA cell death, such as 6-OHDA or MPTP, will be important to validate this hypothesis.

### Relationship between T_2 _relaxivity changes detected by MRI with behavioural and histological outcomes

In contrast to our findings, previous studies have identified increased T_2 _relaxation times, visualised as areas of signal hyperintensity on T2W MR images in both 6-OHDA-lesioned rodents [27] and MPTP-treated primates [28]. Importantly, these findings were reported at acute time-points post-lesion (24 hrs to 7 days) and T_2 _hyperintense areas were observed to dissipate on subsequent MRI scans with increasing time post-lesion [27,28]. It is therefore possible that T_2 _relaxation may evolve with time, although this may not be a reflection of a continuous physiological phenomenon. Indeed, increases in T_2 _at acute stages post-lesion most likely reflect acute oedema [27]. By contrast, at longer time-points post-lesion, iron accumulation appears to be the primary cause of T_2 _signal decrease, as suggested by our findings and those of others [30,46]. Importantly, although previous studies have suggested a link between changes in SN T_2 _relaxation time and nigral cell death [27], this has not been robustly established *in vivo*. We, for instance, could not find a significant relationship between ipsilateral SN T_2 _values and either TH+ or NeuN+ cell number in the SNc. Although the number of subjects in our study was small, these data are in agreement with recently published findings in MPTP-treated primates [30]. One plausible explanation of these findings may be related to iron accumulation in the SN. Iron deposition is known to affect MR signal intensity by creating magnetic field inhomogeneities that de-phase nearby water-protons leading to shortening of T_2 _relaxation time and signal drop off in the affected tissues [47]. In support of this, we observed substantial iron accumulation in the ipsilateral SN of lactacystin-lesioned animals, but not saline controls. Furthermore, we observed a significant relationship between T_2 _and iron accumulation as measured by Prussian blue histology in the SN 3-weeks post-lesion. These data are in good agreement with the findings of Zhu and colleagues (2007) who initially reported the observation of significant iron accumulation in the SN in this model by biochemical measurement of iron content in the SN [36]. Interestingly, previous studies have shown that treatment with novel iron chelators prevented nigral iron accumulation and protected the nigrostriatal system against lactacystin-induced neurodegeneration *in vivo *[35,36]. Speculatively therefore, *in vivo *MR measurement of nigral T_2 _values may be a potentially useful surrogate measure of the neuroprotective efficacy of novel iron chelators in this rodent model of PD, which may warrant further investigation *in vivo*.

Overall, these data are also consistent with MRI findings in PD patients reporting decreased T_2 _relaxation time in the SN [15-18] and biochemical measurements reporting increased iron accumulation in the SN of PD patients [48,49]. Interestingly, changes in SN T_2 _relaxation in PD patients appear to correlate with some clinical symptoms, although the exact relationship is unclear [19,20]. Based on these clinical reports, it has been suggested that T_2 _signal changes may be a useful surrogate marker of PD progression [19,20]. Importantly, in this PD model at 3 weeks post-lesion, ipsilateral SN T_2 _relaxivity was not significantly associated with any behavioural outcome in this cross-sectional study.

Taken together, it may be suggested that at least two distinct physiological processes underlie changes in nigral T_2_, which evolve over time and may in part counterbalance each other. Clearly therefore, longitudinal studies are required to further elucidate the relationship between changes in T_2 _and behavioural or histological outcomes in this model to determine whether this can be a useful surrogate marker of ongoing nigrostriatal degeneration *in vivo*.

It is also noteworthy, however, that in lactacystin lesioned animals we observed numerous areas of T_2 _hypointensity on our T2W MR images throughout the midbrain and not only in the SN. This finding is inconsistent with the patterns of either iron deposition [48,49] or T_2 _relaxivity changes detected by MRI in PD patients [19,20]. This was reflected in both quantitative T_2 _measurements and post-mortem histology, where clear areas of iron accumulation could be observed not only in the SNc, but also the SNr as well as in areas surrounding the needle tract, where ongoing neuronal loss was also evident.

## Conclusions

In summary, this study has identified a pattern of morphological changes in lactacystin lesioned animals, as well as alterations in nigral T_2 _relaxivity, that are somewhat consistent with published clinical neuroimaging data from iPD patients. Interestingly, morphological, but not T_2 _relaxivity changes, were associated with both behavioural and histological outcomes in this model. This raises the possibility that morphological changes may be non-invasive surrogate markers of nigrostriatal degeneration *in vivo *in this pre-clinical model. However, post-mortem immunohistological analysis revealed that lactacystin induced non-specific neuronal loss in the VM, as well as widespread iron accumulation. Taken together, these data suggest that MRI signal changes in this PD model are not wholly specific to degeneration of the nigrostriatal system alone, potentially limiting the usefulness of these markers. Nevertheless, this model has pathological relevance to human iPD, and may be a useful tool to study the relationship between proteasome dysfunction, abnormal accumulation of α-synuclein and DA neurodegeneration *in vivo*. Moreover, these data reinforce the usefulness of MRI for non-invasive evaluation of pathological changes in the brain in pre-clinical models of neurodegenerative disease.

## Methods

### Experimental animals

Male Sprague-Dawley rats (250 ± 10 g, Harlan, UK) were housed in groups of three at 21 ± 1°C on a 12 hr light: dark cycle (lights on 0700, lights off 1900). Standard rat chow and drinking water were available *ad libitum*. All animal experiments were carried out with local ethical approval and in accordance with the Home Office Animals (Scientific procedures) Act, UK, 1986.

Animals were randomised to either saline or lesion groups (*n *= 6 per group) and anaesthetised by i.p. injection of a mixture of medetomidine hydrochloride (Domitor®, 0.25 mg/kg) and ketamine hydrochloride (Vetalar™, 230 mg/kg) in sterile water and positioned in a stereotaxic frame (Kopf Instruments, Tujunga, CA, USA) with the incisor bar set 3.3 mm below the interaural line. Nigrostriatal lesions were induced by a unilateral intranigral injection of the proteasome inhibitor lactacystin (10 μg in 2.5 μl; L6785, Sigma-Aldrich, Poole, UK) at the following stereotaxic co-ordinates, AP: -5.2 mm, ML: +2.4 mm lateral from bregma and -7.6 mm ventral to dura [50] as previously described [34]. Lactacystin was dissolved in 0.9% saline (pH 7.4) immediately prior to use and stored on ice to prevent degradation. Injections were performed at a rate of 1 μl/min using a motorized syringe pump and the needle was slowly withdrawn 5 min after lesioning to minimize diffusion of toxin into the injection tract. Anesthesia was reversed 1 hr after induction by subcutaneous (s.c.) injection of atipamezole hydrochloride (Antisedan®, 5 mg/kg). Sham-lesioned animals underwent identical surgery, but received an injection of 0.9% saline. Saline and lactacystin groups were operated on in a randomised fashion in the same surgical session. Post-operative care included analgesia (buphrenorphine, 0.3 mg/kg s.c. during the first 48 hr), fluid-replacement (4 ml glucosaline solution i.p.) and mashed high-nutrient food pellets during the first week after surgery. Animals were weighed and semi-quantitatively scored daily for neurological deficits using a general neurological rating scale as previously described [51]. This was done daily in a blinded fashion until the end of the experiment.

### Behavioural testing

Animals were routinely handled prior to and after surgery in order to calm the animals and enhance reliability when testing. Prior to any behavioural testing (training or trial) animals were allowed to acclimatize to the testing room for 30 min.

#### Grip strength meter test

To investigate forelimb motor dysfunction following lactacystin-lesioning performance in the grip strength test was assessed using a grip strength meter (GSM; TSE Systems, Bad Homberg, GER) as previously described [52]. Briefly, forelimb grip strength was tested in sessions consisting of five trials separated by approx. 1 min. Animals underwent training prior to surgery to familiarise themselves with the GSM apparatus. This consisted of one day of testing for five trials, per paw, per animal. Subsequent test sessions were conducted preoperatively to establish a baseline, with subsequent re-testing 3 weeks post-surgery. Test sessions were performed on one day both in the morning and afternoon, as per training with 5 trials per paw, per animal. The mean force exerted on the GSM by each forepaw just prior to grip release was recorded automatically per paw (contralateral and ipsilateral to the lesion) for each animal by a strain gauge connected to a digital readout. An average grip force was then calculated for each limb, for all animals in each group.

#### Rotational asymmetry

Three weeks post-lesion, drug-induced rotation as an index of nigrostriatal damage was evaluated in a bank of eight rotameter bowls. Lesioned and control animals were harnessed into jackets tethered to an automated rotameter and allowed to acclimatize for 30 min before administration of apomorphine hydrochloride (0.1 mg/kg dissolved in 0.9% saline, s.c., Sigma-Aldrich, Poole, UK). The numbers of complete contraversive rotations were then measured over 60 minutes using an automated rotameter (TSE Systems, Bad Homberg, Germany).

### Magnetic resonance imaging

#### Preparation

Animals underwent MRI imaging 3 weeks post-surgery, after completion of behavioural testing, based on previous behavioural and histological studies of nigrostriatal damage in this model [34]. Animals were anesthetized using isoflurane (5% induction, 1.5% maintenance) in an oxygen/medical air (30%: 70%) mixture delivered at 1 L/min and placed in a PTFE MRI compatible stereotaxic head frame to reduce head movement. Body temperature, respiration rate, blood oxygen saturation and pulse rate were monitored for the duration of the scan. Animal body temperature was maintained at 37°C using a heated blanket.

#### Image acquisition

T2W MR images were acquired using a 7.0 T horizontal small bore magnet (Varian, Palo Alto, CA, USA) and a custom built head RF coil (David Herlihy) linked to a LINUX-based control console running VnmrJ acquisition software (v2.3, Varian, Palo Alto CA, USA). T2W images were acquired using a multi-echo, multi-slice spin-echo pulse sequence (MEMS), with the following scan parameters: FOV = 35 mm × 35 mm; matrix = 192 × 192; *TR *= 4200 ms; *TE *= 10, 20, 30, 40, 50, 60, 70, 80 ms; 4 averages, scan duration 54 minutes. Fifty contiguous 500 μm-thick coronal slices with an in plane resolution of 189 × 189 μm were acquired such that the entire brain of each animal was covered. Once scanning was completed, animals were removed from the magnet bore to a separate holding room, administered 4 ml 0.9% saline solution (i.p.) and placed in a heated recovery chamber. Following full recovery from anaesthesia, animals were then returned to their home cages.

#### Image post-processing

Brain images were transferred from the console to a workstation, where they were visually inspected prior to post-processing for motion or intensity artefacts. No scans were excluded on this basis. Images were interpolated from the acquired 192 × 192 matrix corresponding to a 189 μm in-plane linear pixel size to a 256 × 256 pixel grid (136 μm voxel size). For quantitative manual volumetric measurements, images corresponding to TE = 10-80 ms were summed and converted to Analyse 7.5 (Mayo Clinic). Quantitative T_2 _relaxation maps were also obtained by a mono-exponential fit of the eight multi-echo images (TE = 10-80 ms) using VnmrJ software.

#### MR image analysis

For whole brain and regional volumetric analysis, structures were delineated manually. Individual brain structures were delineated by a single reviewer (A.C.V) blinded to animal surgical status on a slice-by-slice basis in the coronal plane using the ROI tool in JIM v5.0 software (Xinapse systems, Thorpe Waterville, UK). ROIs were traced in both the ipsilateral and contralateral hemispheres of sham and lesioned animals at low magnification followed by manual correction of borders at higher magnification. Clear anatomical landmarks and reference to the standard rodent brain atlas [50] were used to define ROIs for each region analysed in each slice (Table 1). Volumes were calculated by multiplying the sum of the areas of a given structure on all slices by the slice thickness for each animal. Intra-rater-reliability was assessed following repeated measurements using the intra-class-correlation coefficient (ICC). For all brain regions examined, an ICC value of ≥ 0.9 was obtained, suggesting robustly reliable segmentation of brain structures.

Intensity values for T_2 _were measured in both the contralateral and ipsilateral hemispheres of saline and lactacystin-lesioned animals in the striatum, ventral midbrain and the SN from quantitative T_2 _maps using VnmrJ software. To measure T_2_, an ROI was drawn using the ROI tool in VnmrJ on a representative coronal slice, examples of which are shown for each brain region analysed in Figure 4B,D and 4F. A rodent brain atlas [50] was used to interpret the MR images and define ROI borders. For the SN, atlas plates containing the SN were overlaid onto MR images to aid identification of this structure. No difference could be made between the pars reticulata and the pars compacta of the SN thus T_2 _measurements in this area refer to the whole SN. Values for T_2 _are expressed as the ratio between the contralateral and ipsilateral striatum and SN (relative T_2_).

### Tissue collection

One day post-imaging, animals were terminally anaesthetised by a sodium pentobarbital overdose (60 mg/kg i.p.) and transcardially perfused first with 0.9% saline, followed by ice-cold 4% paraformaldehyde (PFA) in 0.2 M phosphate buffer, pH 7.4. Brains were rapidly dissected out, post-fixed for 24 hours and cryo-protected in buffered 30% sucrose. Serial coronal sections (40 μm) were cut on a freezing microtome at -20°C and stored in cryoprotective solution containing 0.05% sodium azide at -20°C until processed for immunohistochemistry.

### Immunohistochemistry

Free-floating sections were immunostained with the following primary antibodies, rabbit α-tyrosine hydroxylase (TH, AB151, Chemicon, 1:3000), mouse α-Neuron specific nuclear protein (NeuN, MAB377, Chemicon, 1:1000), mouse anti-α-synuclein (610786, BD Biosciences, 1:100) and rabbit α-dopamine and cAMP-regulated phosphoprotein-32 (DARPP-32; AB1656; Chemicon, 1:1000) using a standard immunoperoxidase method as described elsewhere [53].

Briefly, sections were rinsed with PBS, then incubated for 10 min in 0.3% H_2_O_2 _(Sigma-Aldrich) to quench endogenous peroxidase activity. For α-synuclein immunohistochemistry, sections were then incubated with or without PBS containing 10 μg/ml proteinase K (Promega, Southampton, UK) for 10 min at room temperature, to visualise both soluble and insoluble α-synuclein. Non-specific binding was blocked by incubation for 1 hr in blocking solution containing 10% normal sera and 0.03% triton X-100 diluted in PBS. All antibodies were diluted in blocking solution and sections were thoroughly washed in PBS between each step. Tissue sections were incubated with primary antibodies at 4°C overnight, followed by 2 hr incubation at room temperature (RT) with secondary biotinylated anti-mouse antibodies (1:200, Vector Laboratories) and a further 1 hr incubation at RT with an avidin-biotinylated-peroxidase complex (Vectastain ABC kit, 1:100 in PBS, Vector Laboratories). The substrate 3,3'-diaminobenzidine (DAB Fast tablets, Sigma-Aldrich, dissolved in dH_2_O with the addition of H_2_O_2 _to a concentration of 0.03% immediately before use) was used as chromagen. Antibody specificity was confirmed in adjacent tissue sections with the primary or secondary antibody omitted.

Additional nigral and striatal sections were processed for iron accumulation using Pearl Prussian blue staining as described elsewhere [46]. Finally, sections were washed in PBS, counterstained with cresyl fast violet (CFV) and mounted onto glass slides, dehydrated in graded alcohol and xylene before being coverslipped with DPX (Merck, Lutterworth, UK).

### Unbiased stereology cell counts of nigral dopaminergic neurons

To estimate the remaining number of TH-positive (TH+) neurons within the SN in sham and lactacystin-lesioned animals, cell counts were performed using an unbiased stereological three-level fractionator sampling method on a computerized image analysis set-up (Zeiss Axioscope, Carl Zeiss, Gottingen, GER) running Stereo investigator software (v7.0, MBF Bioscience, Chicago, IL, USA). Every 6^th ^section throughout the rostro-caudal extent of the SN was systematically sampled using an unbiased counting frame (the "optical fractionator") of known area superimposed on the field of view by the software. In each tissue section analyzed, section thickness was assessed empirically and guard zones of 2 μm thickness were used at the top and bottom of each section. The SN was manually outlined at ×2.5 magnification and counting frames were systematically distributed with known x and y steps throughout the region from a random starting point. At least 20 counting frames were sampled per section. All cell counts were performed under ×40 magnifications. The coefficients of error (CE) were calculated according to the procedure of West and colleagues with values <0.10 accepted [54]. To ensure the absence of bias in cell counting, slides were coded such that the operator was blinded to the surgical status of the animal. To establish that changes in TH+ cells reflected cell death and not reduced expression of TH protein, additional cell counts were performed in parallel for NeuN-positive (NeuN+) cells in the SN as described above.

### Optical density measurements

Optical density of TH+ fibres in the corpus striatum was measured as previously described with modification [55]. Briefly, digital images of TH-stained sections were captured in a blinded fashion from coded slides across 8 different rostral to caudal levels of the striatum (+1.5 to -2.0 mm AP relative to bregma) at ×2.5 magnification using a Zeiss Axioscope microscope. At each level, for both the ipsilateral and contralateral hemispheres of the brain, images were manually thresholded to highlight TH-staining in the striatum. The area containing the striatum in each section was then traced manually and optical density automatically calculated using the area fraction tool in ImageJ software (NIH, http://rsb.info.nih.gov/ij/). Non-specific background densities were measured in the corpus callosum (an area devoid of TH staining) and subtracted to give corrected striatal optical density values. This method was also applied to quantify the degree of iron deposition in the SN. Briefly, digital images of tissue sections stained with Prussian blue and nissl stain were captured from coded slides at × 40 magnifications using a Zeiss Axioscope microscope. The optical density of Prussian blue stained cells was quantified as outlined above, from 3-5 sections per subject in each group.

### Statistical analysis

All data are expressed as the mean ± SEM. Net contraversive turns for lactacystin or saline-injected treated animals in response to apomorphine hydrochloride were plotted against time and the area under the curve (AUC) calculated in Graphpad Prism (v4.0 San Diego, CA, USA). The resultant means of AUC ± SEM for lactacystin or saline-injected animals were then compared using two-tailed paired student's t-test. Grip strength data, MR regional volumes, quantitative T_2 _measurements and histological data were analysed using standard paired two-tailed student t-tests to compare differences within groups and unpaired two-tailed t-tests to compare differences between groups in Graphpad Prism. Correlations between regional brain volume changes, histology and motor behaviour were calculated for all subjects (both saline controls and lactacystin-lesioned animals) using a two-tailed Pearson correlation analysis in SPSS (v16.0, Woking, UK). In all cases, statistical significance was set at *p *< 0.05.

## Authors' contributions

ACV participated in the design of the study, carried out *in vivo *MR imaging, MR image analysis, behavioural testing, immunohistochemistry, stereology cell counting, optical density measurements, statistical analysis and drafted the manuscript. SJ assisted with surgery, stereology, cell counting, and optical density measurements and helped to draft the manuscript. MM conceived of the study, participated in its design and helped to draft the manuscript. All authors read and approved the final manuscript.

## Acknowledgements

This study was supported by a grant from the Edmond J. Safra philanthropic foundation, which we thank for their generous financial assistance. We also thank the British Heart Foundation for supporting the 7 T MRI scanner (Preclinical imaging unit, Kings College London) used in this study. ACV is supported by an Edmond J Safra fellowship. MM is supported by an RCUK Fellowship.
